# Utilization of Immunotherapy as a Neoadjuvant Therapy for Liver Transplant Recipients with Hepatocellular Carcinoma

**DOI:** 10.3390/jcm13113068

**Published:** 2024-05-24

**Authors:** Maen Abdelrahim, Abdullah Esmail, Mukul K. Divatia, Jiaqiong Xu, Sudha Kodali, David W. Victor, Elizabeth Brombosz, Ashton A. Connor, Ashish Saharia, Ahmed Elaileh, Ahmed O. Kaseb, Rafik Mark Ghobrial

**Affiliations:** 1 Section of GI Oncology, Department of Medical Oncology, Houston Methodist Neal Cancer Center, Houston Meth-Odist Hospital, Houston, TX 77030, USA; aesmail@houstonmethodist.org (A.E.);; 2 Cockrell Center of Advanced Therapeutics Phase I Program, Houston Methodist Research Institute, Houston, TX 77030, USA; 3 Department of Medicine, Weill Cornell Medical College, New York, NY 10065, USA; 4 Department of Pathology and Genomic Medicine, Houston Methodist Hospital, Houston, TX 77030, USA; 5 Lynda K. and David M. Underwood Center for Digestive Disorders, Department of Medicine, Houston Methodist Hospital, Houston, TX 77030, USA; 6 Sherrie and Alan Conover Center for Liver Disease and Transplantation, JC Walter Jr. Center for Transplantation, Houston Methodist Hospital, Houston, TX 77030, USA; 7 Department of Surgery, Houston Methodist Hospital, Houston, TX 77030, USA; 8 Department of Surgery, Weill Cornell Medical College, New York, NY 10065, USA; 9 Department of Gastrointestinal (GI) Medical Oncology, Division of Cancer Medicine, The University of Texas MD Anderson Cancer Center, Houston, TX 77030, USA

**Keywords:** immunotherapy, neoadjuvant, liver transplant, hepatocellular carcinoma, Milan criteria

## Abstract

**Background:** Hepatocellular carcinoma (HCC) is widely recognized as the predominant type of primary liver malignancy. Orthotopic liver transplantation (OLT) has emerged as a highly effective treatment option for unresectable HCC. Immunotherapies as neoadjuvant options are now being actively investigated in the transplant oncology era to enhance outcomes in patients with HCC. Here, we report our experience with patients with HCC who had received Immune Checkpoint Inhibitors (ICPI) prior to curative OLT. **Methods:** This was a retrospective cohort that included patients with HCC who received ICPI prior to OLT at a single institution from January 2019 to August 2023. Graft rejection was assessed and reported along with the type of ICPI, malignancy treated, and the timing of ICPI in association with OLT. **Results:** During this cohort period, six patients with HCC underwent OLT after neoadjuvant ICPI. All patients were male with a median age of 61 (interquartile range: 59–64) years at OLT. Etiology associated with HCC was viral (*N* = 4) or Non-alcoholic steatohepatitis, NASH (*N* = 2). Tumor focality was multifocal (*N* = 4) and unifocal (*N* = 2). Lymphovascular invasion was identified in four patients. No perineural invasion was identified in any of the patients. All patients received ICPI including atezolizumab/bevacizumab (*N* = 4), nivolumab/ipilimumab (*N* = 1), and nivolumab as monotherapy (*N* = 1). All patients received either single or combined liver-directed/locoregional therapy, including transarterial chemoembolization (TACE), Yttrium-90 (Y90), stereotactic body radiotherapy (SBRT), and radiofrequency ablation (RFA). The median washout period was 5 months. All patients responded to ICPI and achieved a safe and successful OLT. All patients received tacrolimus plus mycophenolate as immunosuppressant (IS) therapy post-OLT and one patient received prednisone as additional IS. No patient had clinical evidence of rejection. **Conclusions:** This cohort emphasizes the success of tumor downstaging by ICPI for OLT when employed as the neoadjuvant therapy strategy. In addition, this study illustrated the importance of timing for the administration of ICPI before OLT. Given the lack of conclusive evidence in this therapeutic area, we believe that our study lays the groundwork for prospective trials to further examine the impact of ICPI prior to OLT.

## 1. Introduction

Hepatocellular carcinoma (HCC) is widely recognized as the predominant form of primary liver tumor, with an estimated annual incidence of 2–4% in the United States [1]. Multiple risk factors contribute to the development of this disease, including hepatitis B and C, alcohol use disorder, high-fat diets, biliary cirrhosis, exposure to food toxins such as aflatoxins, and inborn errors of metabolism such as alpha-1 antitrypsin deficiency, hemochromatosis, and Wilson’s disease [2]. HCC is currently the fifth most common type of cancer worldwide and the second leading cause of cancer-related mortality in men, after lung cancer [3]. The 5-year survival rate for HCC is only 18% [4]. 

Orthotopic liver transplantation (OLT) has emerged as a highly effective treatment option for unresectable HCC in patients meeting the Milan criteria, which specify a maximum tumor diameter of 5 cm for a single lesion or no more than three tumors, each less than or equal to 3 cm, without vascular invasion or extrahepatic metastases [5,6,7]. However, in the US, the United Network for Organ Sharing (UNOS) exception points are available to patients within Milan or to those whose tumors have been successfully downstaged and are subsequently eligible for deceased donor allocation. Among the various therapies, neoadjuvant immunotherapy has shown promising results in downstaging liver malignancies and limiting their growth. 

Atezolizumab, an immunomodulatory agent, functions by inhibiting the interaction of programmed cell death protein 1 (PD-1) and CD80 receptors (B7-1Rs) with programmed death-ligand 1 (PD-L1), which is highly expressed in certain tumors and is associated with the reduced activation of immune cells, particularly cytotoxic T cells [8]. Nivolumab, a human immunoglobulin G (IgG_) 4-monoclonal antibody, inhibits PD-1, a cell-surface receptor expressed on activated T cells that play a critical role in regulating the immune response [9]. Ipilimumab is a monoclonal antibody agent that activates the immune system by targeting the cytotoxic T-lymphocyte-associated protein 4 (CTLA-4), a protein receptor that downregulates the immune system [10]. The US Food and Drug Administration (FDA) evaluated the safety and effectiveness of nivolumab in combination with ipilimumab for treating HCC, primarily relying on single-arm data from cohort 4 of CheckMate 040 [11]. Nonetheless, the FDA considered an objective response rate (ORR) of 32% with a duration of response (DoR) of 17.5 months as evidence that the nivolumab-ipilimumab combination had an advantage over the available therapy, which had an ORR ranging from 4% to 7%.

Moreover, utilizing immunotherapy and vascular endothelial growth factor (VEGF) inhibitors has improved patient outcomes, as shown by the results of the IMbrave 150 trial [12]. The trial demonstrated that atezolizumab-bevacizumab resulted in overall survival rates of 67.2% (95% CI, 61.3–73.1) at 12 months, whereas sorafenib only had a rate of 54.6% (95% CI, 45.2–64.0). Nonetheless, the possibility of graft rejection remains a primary concern with immunotherapy. Therefore, a washout period must be considered to minimize the risk of adverse events.

This study reports the safety of using different neoadjuvant regimens of immunotherapies, including atezolizumab in combination with bevacizumab, nivolumab as a monotherapy, or ipilimumab for HCC downstaging prior to OLT.

## 2. Methods

This was a retrospective cohort that included patients with HCC who received ICPI prior to OLT at a single institution of Houston Methodist Hospital from January 2019 to August 2023. Graft rejection was assessed and reported along with the type of ICPI, the malignancy treated, and the timing of ICPI in association with OLT.

Liver transplantation was provided to eligible patients who had confirmed HCC diagnosis with histopathological biopsy. Patients had to demonstrate six months of disease stability or tumor regression during neoadjuvant therapy. All patients had to pass through the tumor board that conducts a multidisciplinary case assessment with a view of GI oncologists, transplant surgeons, radiation oncologists, hepatologists, pathologists, and interventional radiologists. Following examination by Houston Methodist Hospital’s GI oncologists and liver transplant surgeons, a majority judgment was made about the viability of tumor resections. In addition, patients had to go through a full medical and psychological evaluation. After that, candidates went for a formal liver transplant evaluation, and a listing was presented. Patients were required to undergo neoadjuvant therapy for six months in order for their tumors to remain unresectable, either because of the tumor’s location or underlying liver disease, as shown by a biopsy or cytology. The study’s protocol was approved by the Houston Methodist Institutional Review Board (IRB ID: PRO00000587).

Patients were followed up on an eight-week basis to assess the therapy response with imaging such as liver contrast enhancement in computed tomography (CT) or magnetic resonance imaging (MRI). In addition, complete blood cell count, liver function tests of aspartate aminotransferase (AST), alanine aminotransferase (ALT) and bilirubin, albumin, as well as alpha-fetoprotein test (AFP). The Modified Response Evaluation Criteria in Solid Tumors (mRECIST) was utilized to evaluate the tumor response.

From the statistical analysis standpoint, this is a descriptive study. All data were presented as median (IQR) for continuous measures, and n (%) for categorical measures. All analyses were performed with STATA version 17 (StataCorp. 2021. Stata Statistical Software: Release 17. College Station, TX: StataCorp LLC) (Table 1).

## 3. Results

**Figure 1:**
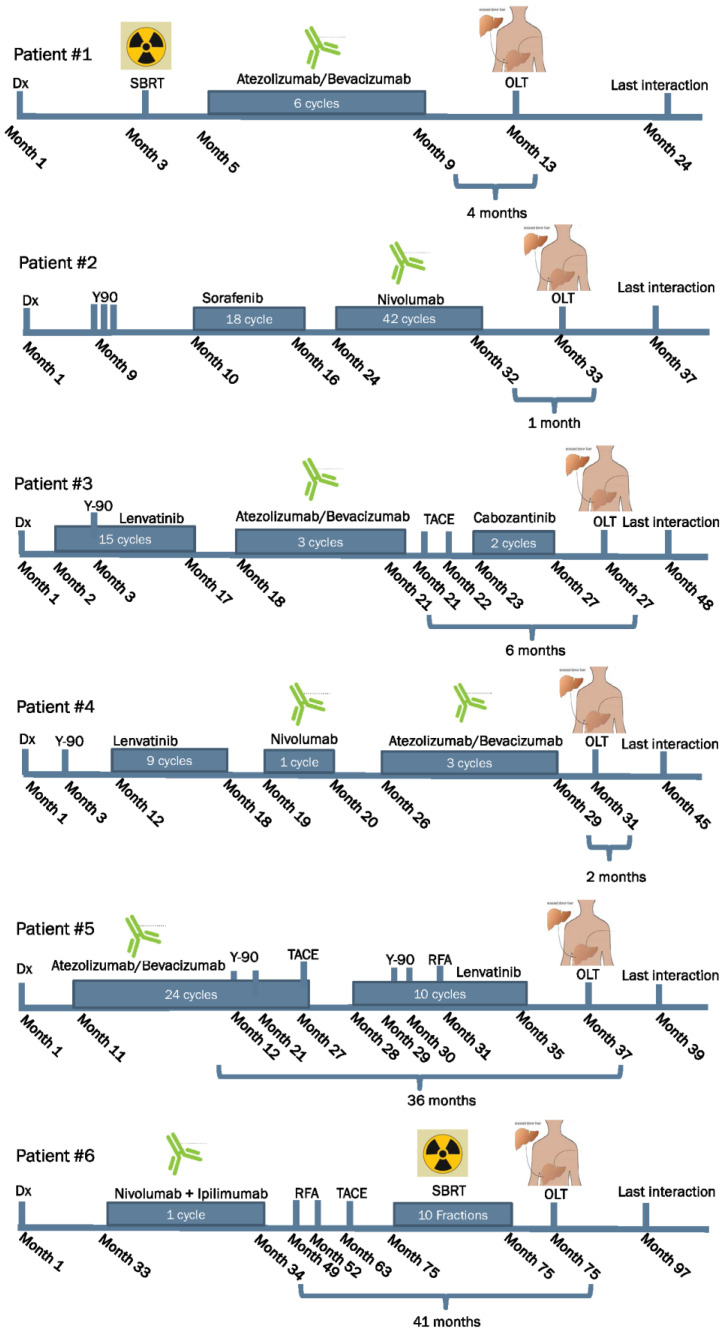
Patient clinical history timeline. TACE: Transarterial Chemoembolization, RFA: Radiofrequency ablation, OLT: Orthotopic liver transplantation, Dx: Diagnosis, SBRT: Stereotactic Body Radiation Therapy.

**Figure 2:**
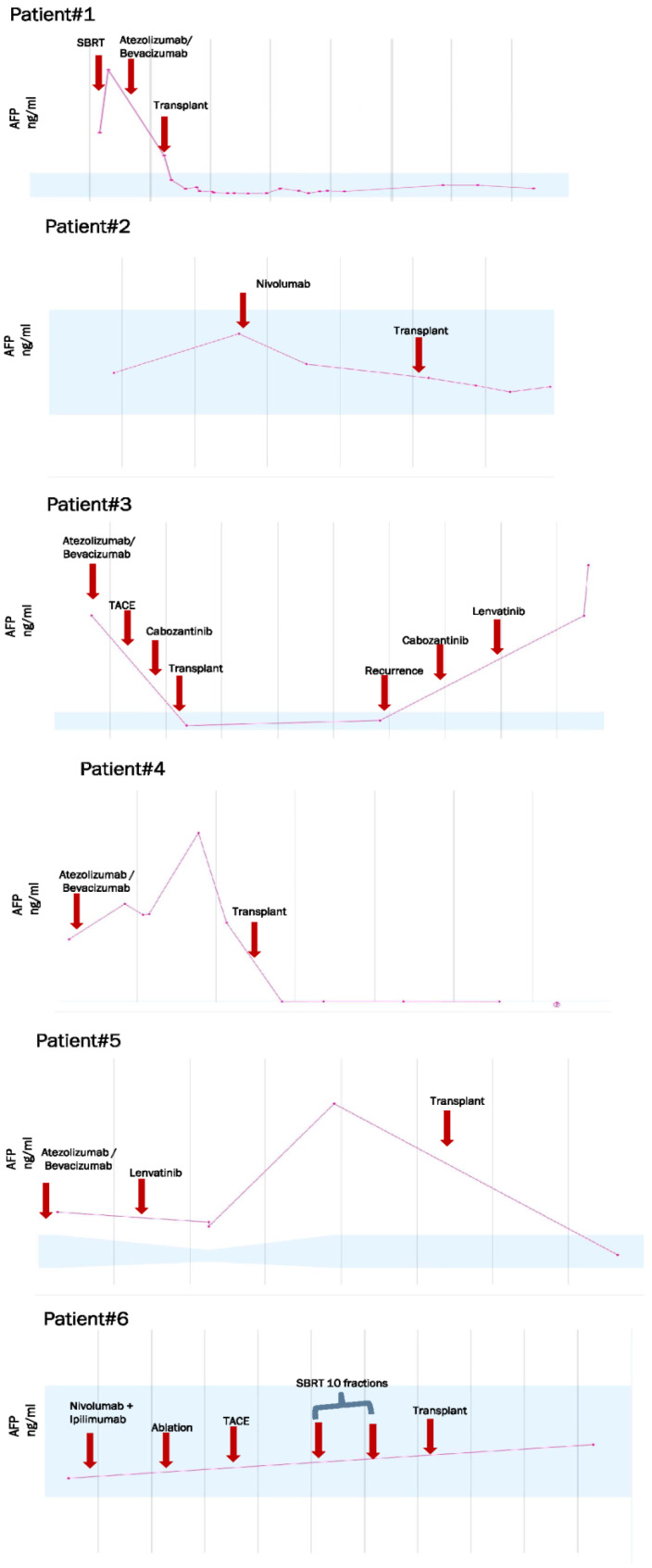
Patient alpha-fetoprotein (AFP) timelines. TACE: Transarterial Chemoembolization, SBRT: Stereotactic Body Radiation Therapy.

Patient 1 was a 64-year-old male with a history of hepatitis C and cirrhosis with ascites, jaundice, encephalopathy, Child C, and a Model for End-Stage Liver Disease (MELD)Na score of 19. He presented with abdominal pain and was found to have a 3.3 cm Liver Reporting & Data System (LI-RADS 5) liver mass. Upon further evaluation, the patient was diagnosed with Stage II Grade 3, poorly differentiated HCC. Within 4 months of diagnosis, the patient received a 4-month course of atezolizumab 1200 mg/bevacizumab 1400 mg. Magnetic resonance imaging (MRI) showed a significant interval treatment response, with a decrease in the size of the mass in segment 7. One year after the initial diagnosis, the patient underwent an OLT. The patient received mycophenolate 500 mg and tacrolimus (0.5 mg) twice daily (BID) for immunosuppression with tacrolimus escalated from 2 to 4 mg and then decreased back to 1 mg. However, one year after the transplant, the patient exhibited symptoms of abdominal pain, anorexia, and uremia. The evaluation revealed occlusion of the intrahepatic inferior vena cava due to the adjacent soft tissue, suggestive of HCC recurrence. The patient’s condition continued to worsen; complications included septic shock secondary to pneumonia (possibly *Pseudomonas*), but there was no clinical evidence of rejection (Figure 1 and Figure 2).

Patient 2 was a 61-year-old male diagnosed with grade 1 Stage IA locally advanced HCC with LI-RADS 5 bilobar hepatic masses. The right-sided lesion was 15 cm in size, with right portal vein invasion. During the following year, the hepatic metastases, hepatic cirrhosis, fatty metamorphosis, and splenomegaly progressed. Therefore, the patient underwent three rounds of yttrium-90 (Y90) therapy until the lesions stabilized, 18 months after diagnosis. The patient underwent a partial hepatectomy. He received bridging therapy with 100 mg nivolumab with an 8-week washout period before OLT. The patient also underwent transarterial chemoembolization (TACE) 2 weeks before transplantation. Explant pathology revealed that the patient had multiple liver lesions, the largest of which was 1.4 cm in diameter. He underwent OLT 33 months after diagnosis. He received 200 mg of mycophenolate and tacrolimus (0.5 mg) daily, escalating to 3 mg. The patient showed no clinical evidence of rejection (Figure 1 and Figure 2). 

Patient 3 was a 58-year-old male who presented with a history of chronic hepatitis B and elevated alpha-fetoprotein (AFP) levels, which prompted further evaluation. He was diagnosed with stage IV LI-RADS 4–5 multifocal HCC, which was confirmed by wedge liver biopsy. Within a month of diagnosis, the patient started receiving 8 mg of lenvatinib and also received right-sided Y90 2 months after diagnosis. He initially showed a significant response; however, 10 months after diagnosis, magnetic resonance imaging (MRI) revealed an increase in the prominence of the liver lesion. In response, the patient was switched to 1200 mg atezolizumab and 1400 mg bevacizumab, but still showed significant disease progression with no improvement. Three months after atezolizumab/bevacizumab administration, the patient underwent right- and left-sided TACE; left-side TACE showed a good response at that time. Accordingly, a new regimen of 40 mg cabozantinib was initiated 4 months prior to OLT. He underwent OLT 2.3 years after his initial diagnosis, after which he received 80 mg regorafenib as a systemic adjuvant therapy for 1-year post-OLT. The patient also received 1000 mg of mycophenolate and 0.5 mg of tacrolimus BID as immunosuppressants. Subsequently, MRI revealed new multiple enhancing lesions in liver segments 6 and 8, and new enlarging pulmonary nodules were identified on chest computed tomography (CT). After these scans, the patient was started on 40 mg cabozantinib for a month and 8 mg lenvatinib for 3 months. Alternative therapies are currently being explored for patient treatment. Importantly, despite the multiple neoadjuvant and adjuvant immune checkpoint inhibitor therapies received by this patient, there was no clinical evidence of graft rejection (Figure 1 and Figure 2).

Patient 4 was a 61-year-old man who presented to the hospital with abdominal pain and bloating and was found to have a 1.9 cm lesion compatible with HCC and a suspicious 1.4 cm new lesion. The tumor was confined to the liver with clear margins, no vascular invasion, normal regional lymph nodes according to the imaging criteria, and no evidence of distant metastasis. Liver enzyme and AFP levels were elevated at the time of presentation and returned to normal by the end of management. The patient had a medical history of hemochromatosis, cirrhosis, non-alcoholic steatohepatitis, and hemosiderosis, with +4 iron staining in hepatocytes. The patient also experienced acquired pancytopenia, portal hypertension, and portal vein thrombosis. The patient had a 2.1 cm LI-RADS 5 lesion two months before starting immunotherapy. He was started on lenvatinib 1 year and 7 months after diagnosis but discontinued because of intolerance and worsening bilateral leg ulcers. One month later, the patient began a nivolumab regimen (240 mg) that was well tolerated. However, the patient’s HCC progressed, and he was switched to 1200 mg atezolizumab and 1400 mg bevacizumab for 2 months with a 3-month washout period prior to OLT, which he received 2.5 years after diagnosis. The patient received mycophenolate 500 mg twice daily (BID), tacrolimus 3 mg BID, and everolimus 1.5 mg BID. One year after the OLT, abdominal/pelvic MRI, and chest CT showed no evidence of relapsed cancer, and no clinical rejection was observed (Figure 1 and Figure 2). 

Patient 5 was a 68-year-old male who presented with Stage IIIB HCC (cT4, cN0, and cM0). Multiple bilobar lesions were identified via MRI. The largest discrete mass (7.5 cm) was a LI-RADS 5 lesion in segments 5/6 with heterogeneous enhancement, delayed washout, and a pseudocapsule. Additionally, smaller hypervascular lesions were observed. Two months after diagnosis, the patient was started on 1400 mg bevacizumab and 1200 mg atezolizumab, which was continued for 15 months but discontinued due to disease progression. The patient had undergone multiple Y90 procedures. One month after discontinuing atezolizumab and bevacizumab, the patient started oral lenvatinib 8 mg/day and had excellent tolerance. Imaging 5 months pre-OLT showed residual disease in segment 4 and a stable LI-RADS 3 hypervascular nodule in segment 8, with stable lesions elsewhere. The pre-transplant period was complicated by bacteremia, septic shock, renal failure, and acute respiratory failure, all of which were medically treated. The patient underwent OLT 2.5 years after the initial diagnosis. The patient received 500 mg of mycophenolate and tacrolimus BID (0.5 mg tacrolimus BID, which was increased to 4 mg and then tapered to 0.5 mg. He developed chronic respiratory failure requiring tracheostomy, sustained bacteremia with septic shock, and renal failure requiring dialysis. However, clinical graft rejection was not suspected or observed in our patient (Figure 1 and Figure 2).

Patient 6, a male aged 59 years, had previously been diagnosed with HCC in the context of hepatitis B. HCC was initially resected, but it recurred, and he underwent re-resection 3 years later. He had a cycle of preoperative nivolumab and ipilimumab but experienced presumed immune checkpoint inhibitor (ICPI)-related hepatotoxicity (autoimmune hepatitis) that improved on prednisone (20 mg). Therefore, adjuvant immunotherapy was not recommended. A year after resection, radiologic imaging demonstrated tumor recurrence with a new left anterior hepatic 1.5 cm in diameter, which was treated with radiofrequency ablation shortly thereafter. Subsequent scans over the following three months showed an additional 1.1 cm tumor nodule in close proximity to the prior recurrence, which was also treated with radiofrequency ablation. After nine months, the scans revealed another 2.4 cm in diameter tumor recurrence adjacent to the left portal vein, which was treated with TACE. One year later, imaging studies indicated the presence of a new 3.3 cm LI-RADS 5 lesion that was treated with stereotactic body radiotherapy (SBRT) over the course of 10 days. The patient ultimately underwent OLT two years after the last recurrence. His post-OLT immunosuppression regimen included 10 mg prednisone, 3 mg tacrolimus, and 500 mg mycophenolate 500 mg. Since then, he has been hospitalized twice with mesh-related complications and infection but has no clinical evidence of rejection (Figure 1 and Figure 2). 

The treatment details for this study cohort are summarized in Table 1 and Table 2, and the pathological features identified in the explanted liver specimens examined following orthotopic transplantation are shown in Table 3.

## 4. Discussion

Our study included six patients who received various immunotherapy regimens, and none of them exhibited any clinical evidence of rejection. A combination therapy of atezolizumab and bevacizumab was administered to four of our patients. Patients 1 and 4 in this report were treated with neoadjuvant atezolizumab and bevacizumab combination therapy for HCC of stages II and I in the same order, discontinued in preparation for OLT, which was performed for 4 and 3 months, respectively, following the final administration of immunotherapy. Patient 5, who presented with stage III HCC, initiated a regimen of combined atezolizumab and bevacizumab. However, the marked progression of the disease caused a switch to another FDA-approved regimen: lenvatinib, with discontinuation of administration five months prior to OLT. Similarly, in the case of patient 3, who had stage V HCC, the initiation of atezolizumab and bevacizumab therapy resulted in disease progression. This treatment was followed by targeted therapy with cabozantinib for 4 months before OLT.

This study reports the safety of utilizing immunotherapy, such as atezolizumab, nivolumab, and ipilimumab, prior to OLT while emphasizing the importance of the timing of ICPI administration in relation to OLT. A previously published cohort of eight patients who received ICPIs with subsequent OLT documented an interval period of 4 weeks between the last dose of ICPI and OLT, with only one case of mild biopsy-proven acute rejection (BPAR) [13]. Acute rejection risks might be increased, especially in the case of a short washout period between the last ICPIs and OLT [14]. Our cases suggest that a prolonged period without immunotherapy prior to OLT may be beneficial for ensuring successful transplant outcomes in patients who previously received immunotherapy. The accurate period of washout before OLT has not yet been confirmed, but reports range from 4 to 12 weeks [12,13,14,15], in our institutional experience, the optimal time for a liver transplant is at least 8 weeks between the last dose of ICPIs and OLT, such that the patient is no longer at risk for a majority of liver-related issues, such as liver allograft rejection or toxicity. 

Additionally, the administration of an immunosuppressant peri-transplant further suppresses the immune system, which is boosted by ICPIs. This would protect the graft from the recipient’s own immune system and play a further role in neutralizing the impact of ICPI at a critical period for the new solid organ allograft, as reported in different case reports [16,17]. In the same direction, it is critical to have comprehensive monitoring standers with the highest quality of preparation to manage any adverse events in HCC patients undergoing ICPI and OLT. 

To fully understand the best strategy for using ICPIs in patients awaiting liver transplantation and improve risk prediction while reducing the graft loss rate, prospective clinical trials are needed in this field. Future studies should prospectively observe differences between patients with previous treatment prior to immunotherapy and those for whom immunotherapy was the initial treatment. Notably, immunotherapy may trigger severe graft rejection in some patients by activating the innate immune response. Hence, the timing of immune ICPI administration is a critical factor that must be carefully considered to achieve favorable outcomes. The viability of administering ICPI prior to liver transplantation will be further clarified by ongoing clinical investigations conducted at our institution (NCT05185505) [12].

Immunotherapy is emerging as a treatment approach that provides encouraging prognostic results in patients with advanced HCC, despite a substantial cohort (up to one-third of cases) of patients who do not benefit significantly from this treatment and immune-related adverse events developing in up to one-fourth of these cases [18]. It is currently difficult to predict which patients will benefit from immunotherapy, based on clinical and pathological parameters. There is a dearth of novel predictive biomarkers that can aid in assessing response to immunotherapy and provide guidance for further clinical treatment strategies. Monitoring the levels of serological laboratory markers, such as AFP, and inflammatory markers, such as C-reactive protein, has demonstrated a strong correlation with patient prognosis in cases of advanced HCC in some published studies [19,20].

**Figure 3:**
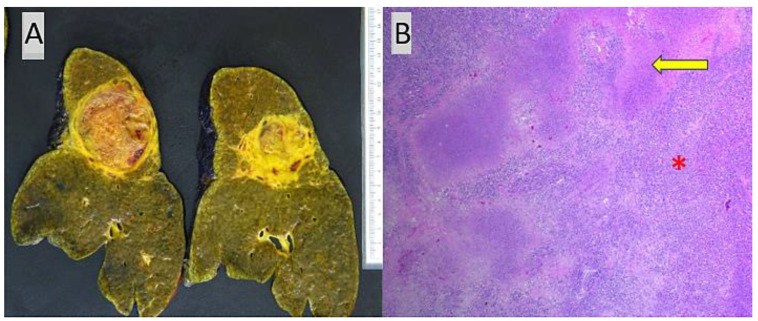
(**A**) Grossly identified necrotic segment 2 tumor nodule with intra-and peritumoral fibrosis in patient #1 following immunotherapy without any prior locoregional treatment, (**B**) corresponding histologic section demonstrating poorly differentiated HCC (*) with geographic zones of tumor necrosis (arrow), H and E stain (×20).

**Figure 4:**
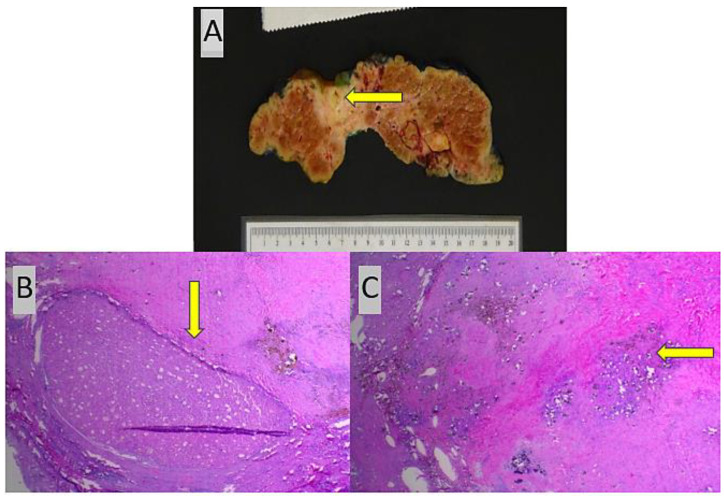
(**A**) Unifocal necrotic tumor nodule (arrow) in segment 4 following both immunotherapy and locoregional treatment in patient #2. (**B**) Single small residual focus of well-differentiated HCC (arrow) spanning 0.4 cm in patient #2, H and E (×20); (**C**) remainder of tumor nodule with extensive treatment-associated necrosis, fibrosis, and numerous Y-90 selective internal radiation treatment beads (arrow), H and E (×20).

Tumor shrinkage or response to treatment was assessed by the relative reduction in tumor size between the baseline diagnosis on imaging and examination of the explanted hepatectomy specimen following locoregional and/or immunotherapeutic treatment. In our study, one of the patients (patient #1) with multifocal HCC did not receive locoregional treatment and yet demonstrated 25% tumor necrosis in a total of three tumor nodules following immunotherapy treatment (Figure 3), which reflects the fact that this modality can also result in significant tumor shrinkage when employed as the sole or first-line treatment strategy. Similarly, patient #2 demonstrated an excellent response to combined immunotherapy and locoregional treatment, resulting in near-complete tumor necrosis with only a single small 0.4 cm focus of well-differentiated HCC identified in the explanted liver (Figure 4).

Overall, the combination of locoregional treatment and immunotherapy resulted in significant tumor necrosis, ranging from 10 to 100 % in all except one case (Table 3). One of the aims of applying the response criteria is to identify patients who are likely to benefit from continuation of the current treatment or patients who are more likely to benefit from alternative treatments when considering an improved overall survival outcome. Considering this, immunotherapy in conjunction with locoregional treatment is additive in reducing the volume of viable tumors, as reflected in the findings of this study. 

While this stay is a retrospective study on a small number of patients, it highlights the significance of timing for ICPI administration before OLT, as well as emphasizing the need for a proper washout period to mitigate the risk of acute rejection. The cohort studies in a prospective manner with high selection criteria are necessary in the future to demonstrate the efficacy of using ICPI agents prior to OLT. In addition, this study encourages identifying proper predictive biomarkers to optimize the patient selection criteria for neoadjuvant ICPI therapy in HCC in an OLT setting.

## 5. Conclusions

Our experience emphasizes the significant tumor downstaging of ICPIs for OLT when employed as the sole or first-line therapy strategy. In addition, this study illustrated the importance of timing for the administration of ICPIs before organ transplantation while highlighting the safety of immunotherapies prior to liver transplantation. Given the lack of conclusive evidence in this therapeutic area, we believe that our study lays the groundwork for prospective trials to further examine the impact of treatment with both CTL-4 and PD-L1 agents prior to OLT. A clinical trial is currently being conducted at our institution to evaluate the feasibility of employing ICPI prior to OLT (NCT05185505). 

## Figures and Tables

**Table 1:** Descriptive statistics: data are presented as median (interquartile range (IQR)) for continuous measures, and n (%) for categorical measures.

**Total: *N* = 6**	
**Age:** 61.00 (59.00–64.00)	
**Sex**	
Male	6 (100.00)
**Ethnicity**	
Hispanic or Latino	1 (16.67)
Not Hispanic or Latino	5 (83.33)
**Race**	
Asian	2 (33.33)
Caucasian	4 (66.67)
**Stage of HCC at the Dx**	
Stage IA	1 (16.67)
Stage II	2 (33.33)
Stage III	1 (16.67)
Stage IIIB	1 (16.67)
Stage IVA	1 (16.67)
**Grade of HCC at the Dx**	
Grade 1	1 (16.67)
Grade 2	2 (33.33)
Grade 2–3	2 (33.33)
Grade 3	1 (16.67)
**Size of Largest HCC lesion (cm) at the Dx** 3.30 (3.00–4.80)	
**ECOG =** 1:6 (100.00)	
**Child–Pugh (CP) class**	
A5	1 (16.67)
A6	1 (16.67)
C10	3 (50.00)
C11	1 (16.67)
**MELD from the Dx to OLT**	
10 to 31	1 (16.67)
13 to 43	1 (16.67)
19 to 30	1 (16.67)
6 to 28	1 (16.67)
6 to 8	1 (16.67)
8 to 43	1 (16.67)
**Comorbidities**	
NASH and Cirrhosis	1 (16.67)
HBV and Cirrhosis	1 (16.67)
HTN, BPH, HCV, and Cirrhosis	1 (16.67)
HTN, DM, HBV, and Cirrhosis	1 (16.67)
HTN, HCV, and Cirrhosis	1 (16.67)
Hemochromatosis and Cirrhosis	1 (16.67)
**AFP (ng/mL) prior OLT:** 21.95 (2.00–53.80)	
**Any treatment prior to ICPI**	
No	4 (66.67)
Yes	2 (33.33)
**Time for neoadjuvant:** 635.50 (518.00–699.00)	

**Table 2:** Basic characteristics of the included patients. ICPI: Immune Checkpoint Inhibitors, PD-1: Programmed Death, PDL-1: Programmed death-ligand 1, CTL-4: cytotoxic T-lymphocyte–associated antigen 4, Dx: Diagnosis, LRT: Locoregional Therapy, Y90: Yttrium-90, OLT: Orthotopic liver transplantation TACE: Transarterial Chemoembolization, HCC: Hepatocellular carcinoma, RFA: Radiofrequency ablation, IS: Immunosuppressant, WOP: Wash out period, AFP: Alpha-fetoprotein, ng/mL: Nanograms per milliliter.

Case ID	Age Years	Gender	Dx	Type of ICPI	LRT	WOP in Months	AFP before OLT	Type of Surgery	Rejection Yes or No	IS Agents after OLT
1	64	Male	HCC	PD-L1	None	4	2.0 ng/mL	OLT	No	Mycophenolate Tacrolimus
2	61	Male	HCC	PD-1	Y-90/TACE	1	1.8 ng/mL	OLT	No	Mycophenolate Tacrolimus
3	58	Male	HCC	PD-L1	TACE/Y-90	6	4.4 ng/mL	OLT	No	Mycophenolate Tacrolimus
4	61	Male	HCC	PD-1 PD-L1	Y-90	2	792.0 ng/mL	OLT	No	Mycophenolate Tacrolimus Everolimus
5	68	Male	HCC	PD-L1	Y-90/ RFA/TACE	36	41.2 ng/mL	OLT	No	Mycophenolate Tacrolimus
6	59	Male	HCC	PD-1 CTLA-4	TACE/RFA	41	2.7 ng/mL	OLT	No	Prednisone Tacrolimus Mycophenolate

**Table 3:** Pathologic findings of explanted total hepatectomy specimens following locoregional treatment and immunotherapy regimens.

Case ID	Tumor Size (cm)	Tumor Focality	Hepatic Segment(s) Involved by HCC	Grade	Tumor Necrosis	Lymphovascular Invasion	Perineural Invasion	Primary Tumor (ypT)	Lymph Nodes	Tumor Stage	Etiology Associated with HCC	Fibrosis Staging
1	3.3, 2.9, 1.2	Multifocal, 3 tumor nodules	2, 2 and 7	Poorly Differentiated	25%	Not identified	Not identified	T2(m)	One (ypN0)	II	Hepatitis B and C	Cirrhosis
2	0.4	Unifocal	4	Well-Differentiated	99%	Not identified	Not identified	T1a	None	IA	Hepatitis C, alpha-1 antitrypsin deficiency	Cirrhosis
3	>20	Multifocal (cirrhotomimetic) >30 tumor nodules	All segments	Moderately Differentiated	20%	Present, small-caliber blood vessels	Not identified	T2(m)	None	II	Hepatitis B	Bridging (stage 3) fibrosis
4	1.2–3	Multifocal, 8 tumor nodules	2, 3, 4, 7 and 8	Moderately to Poorly Differentiated	4 entirely viable tumor nodules, remainder 4 nodules with 10–95% necrosis	Present, small-caliber blood vessels	Present, large- caliber nerve fibers	T2(m)	None	II	NASH, hemochromatosis	Cirrhosis
5	8, 5.2, 2.5	Multifocal, 3 tumor nodules	5/6, 2/4 and 7/8	Moderately to Poorly Differentiated	98% (largest tumor), 70% (second largest tumor), 100% (smallest tumor)	Present, small-and medium-caliber blood vessels	Not identified	T3(m)	Two (ypN0)	IIIA	NASH	Cirrhosis
6	3.4	Unifocal	Perihilar	Well to Moderately Differentiated	Not identified	Present, small-caliber blood vessels	Not identified	T2	None	II	Hepatitis B	Cirrhosis with regression and portal vein thrombosis in Segment 2

## Data Availability

The data of this study that supports our results are available upon request from the corresponding author, Maen Abdelrahim.
